# Phenotypically distinct neutrophils patrol uninfected human and mouse lymph nodes

**DOI:** 10.1073/pnas.1905054116

**Published:** 2019-09-04

**Authors:** Laurence S. C. Lok, Thomas W. Dennison, Krishnaa M. Mahbubani, Kourosh Saeb-Parsy, Edwin R. Chilvers, Menna R. Clatworthy

**Affiliations:** ^a^Molecular Immunity Unit, Department of Medicine, University of Cambridge, Cambridge CB2 0QH, United Kingdom;; ^b^Department of Medicine, University of Cambridge, Cambridge CB2 0QQ, United Kingdom;; ^c^Department of Surgery, University of Cambridge, Cambridge CB2 0QQ, United Kingdom;; ^d^National Institute of Health Research Cambridge Biomedical Research Centre, Cambridge CB2 0QQ, United Kingdom;; ^e^National Heart and Lung Institute, Imperial College London, London W12 0NN, United Kingdom

**Keywords:** neutrophils, lymph node, homeostasis

## Abstract

Neutrophils play a key role in innate immunity. As the dominant circulating phagocyte, they are rapidly recruited from the bloodstream to sites of infection or injury to internalize and destroy microbes. More recently, neutrophils have been identified in uninfected organs, challenging the classical view of their function. Here we show that neutrophils were present in lymph nodes (LNs) in homeostasis. Using flow cytometry and confocal imaging, we identified neutrophils within LNs in naive, unchallenged mice, including LNs draining the skin, lungs, and gastrointestinal tract. Neutrophils were enriched within specific anatomical regions, in the interfollicular zone, a site of T cell activation. Intravital two-photon microscopy demonstrated that LN neutrophils were motile, trafficked into LNs from both blood and tissues via high endothelial venules and afferent lymphatics, respectively, and formed interactions with dendritic cells in LNs. Murine and human LN neutrophils had a distinct phenotype compared with circulating neutrophils, with higher major histocompatibility complex II (MHCII) expression, suggesting a potential role in CD4 T cell activation. Upon ex vivo stimulation with IgG immune complex (IC), neutrophils up-regulated expression of MHCII and costimulatory molecules and increased T cell activation. In vivo, neutrophils were capable of delivering circulating IC to LNs, suggesting a broader functional remit. Overall, our data challenge the perception that neutrophil patrol is limited to the circulation in homeostasis, adding LNs to their routine surveillance territory.

## Significance

The classical view of neutrophils is as circulating phagocytes that are recruited to tissues following infection or injury. Here we show that neutrophils were present in mouse and human lymph nodes in the absence of perturbation. Lymph node neutrophils were phenotypically distinct, with increased expression of major histocompatibility complex II, and predominantly localized to the interfollicular zone, where CD4 T lymphocytes are activated. Neutrophils trafficked into lymph nodes via blood and lymphatic vessels, and were capable of rapidly carrying systemically acquired, IgG antibody-opsonized cargo to lymph nodes. These data support a novel role for neutrophils in homeostatic immune surveillance, sampling circulating antigens and delivering them to lymph nodes, with the potential to activate adaptive immunity.

Neutrophils (polymorphonuclear leukocytes; PMNs) are important effectors of innate immunity. They are produced in bone marrow (BM) (1) and released into intravascular pools (2). Circulating neutrophils, with approximate half-lives of 10 h in mice and 18 h in humans (3–7), can be rapidly recruited to sites of infection or tissue injury (8). To respond to these stimuli, neutrophils express a variety of surface receptors, including Toll-like receptors (TLRs) for direct recognition of pathogen- and damage-associated molecular patterns (PAMPs and DAMPs) (9) and IgG Fc receptors (FcγRs) that mediate internalization of IgG-opsonized pathogens and antigen–antibody immune complex (IC), which may contribute to autoimmunity (10).

There is increasing appreciation of the role of neutrophils in adaptive immunity, including antigen presentation to CD4 T cells via major histocompatibility complex II (MHCII) in vitro (11, 12) and cross-presentation to CD8 T cells via MHCI in vivo (13). Lymph nodes (LNs) are critical sites for T cell activation. T cells traffic into LNs via high endothelial venules (HEVs) dependent on L-selectin/peripheral lymph node addressin (PNAd) (14), stay transiently in search of antigen (15), and reenter circulation via efferent lymphatics. The anatomical arrangement of innate cells within LNs also generates an important barrier against systemic pathogen spread from infected tissues (16, 17). Neutrophils can migrate rapidly to LNs to reinforce this barrier following microbial (18–20) and inflammatory (21) challenges. A recent study has highlighted neutrophils in uninflamed nonlymphoid organs including lung and intestine, suggesting they may contribute to tissue homeostasis (22); whether this occurs in LNs is unclear.

Here we address the question of whether neutrophils traffic tonically into LNs under homeostatic conditions, expanding the anatomical territories they routinely patrol, with the potential to bolster basal cellular innate defense in LNs and influence adaptive immunity by bringing blood-sampled antigen for presentation to CD4 T cells.

## Results

### Neutrophils Are Present in Unstimulated LNs throughout the Body.

**Fig. 1.**
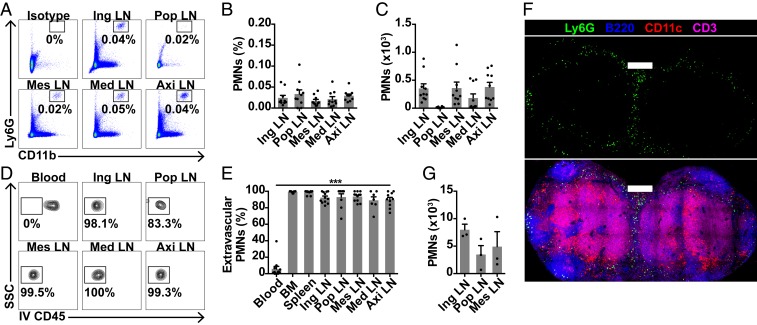
Neutrophils are present in unstimulated LNs throughout the body. (*A*–*C*) Flow cytometry quantification of neutrophils in inguinal (ing), popliteal (pop), mesenteric (mes), mediastinal (med), and axillary (axi) LNs from unstimulated mice, with (*A*) representative plots, (*B*) % live extravascular neutrophils, and (*C*) extravascular neutrophils per LN. (*D* and *E*) Flow cytometry quantification of extravascular neutrophils, with (*D*) representative plots and (*E*) % i.v. CD45^−^ neutrophils. Data are from 12 mice in 9 experiments; ***P < 0.001 across groups. (*F*) Confocal image of an unstimulated C56BL/6 inguinal LN. (Scale bars, 300 μm; Z stack, 5 μm.) (*G*) Confocal quantification of neutrophils in unstimulated LNs; data are from 3 mice. Mean ± SEM is shown.

To address whether neutrophils are present in LNs under homeostatic conditions, we administered i.v. anti-CD45 to wild-type mice to label all circulating leukocytes, and harvested inguinal, popliteal, mesenteric, mediastinal, and axillary LNs. We used young female mice to avoid confounding from combat injuries in cohoused male mice. We observed Ly6G^+^CD11b^+^ neutrophils in all sampled LNs (Fig. 1 *A*–*C* and *SI Appendix*, Fig. S1 *A* and *B*); the vast majority were i.v. anti-CD45–negative (Fig. 1 *D* and *E* and *SI Appendix*, Fig. S1*C*), suggesting these were bona fide interstitial LN neutrophils. Analysis of tissue immune cells by disaggregation and flow cytometry can underestimate cell numbers (23). We therefore performed confocal microscopy, similarly identifying neutrophils within LNs, distributed mainly peripherally, and in interfollicular and interlobar areas (Fig. 1*F* and *SI Appendix*, Fig. S1*D*). Confocal quantification showed true LN neutrophil numbers to be 10-fold higher than flow cytometry estimates (Fig. 1*G*). Together, these data show that neutrophils are present in unstimulated LNs in homeostasis.

### The Majority of LN Neutrophils Are within Tissues and Lie outside Blood and Lymphatic Vessels.

**Fig. 2.**
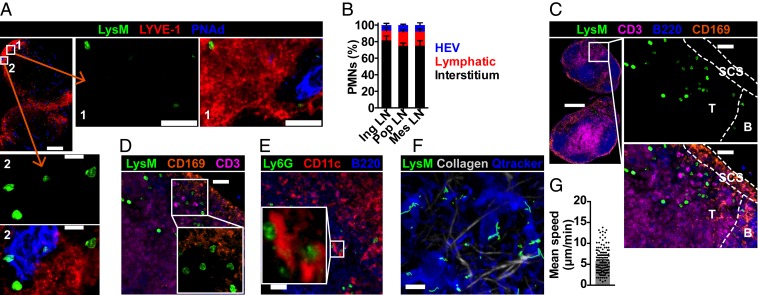
Majority of LN neutrophils are within tissues and lie outside blood and lymphatic vessels. (*A*) Confocal image of unstimulated LysM-GFP inguinal LN. (Scale bars, 300 μm [*Inset* 1, 40 μm; *Inset* 2, 15 μm]; Z stack, 10 μm.) (*B*) Confocal quantification of LN neutrophil location; data are from 3 mice. (*C*) Confocal image of inguinal LN neutrophil location. (Scale bars, 300 μm [*Inset*, 30 μm]; Z stack, 20 μm.) (*D*) Confocal image of inguinal LN showing neutrophils near SCS macrophages. (Scale bar, 30 μm; Z stack, 20 μm.) (*E*) Confocal image of inguinal LN showing neutrophil colocalization with DCs. (Scale bar, 40 μm; Z stack, 11 μm.) (*F*) Intravital imaging of unstimulated LysM-GFP popliteal LN; neutrophil movements are in green tracks. (Scale bar, 40 μm; Z stack, 60 μm.) (*G*) Mean speed of LN neutrophils; data are from 4 movies, each dot representing one cell track. Mean ± SEM is shown.

We next determined the anatomical location of neutrophils. Few neutrophils were within HEVs and lymphatic vessels, identified by PNAd and lymphatic vessel endothelial hyaluronan receptor 1 (LYVE-1), respectively, but the majority (75 to 82%) of neutrophils were within LN interstitium (Fig. 2 *A* and *B* and Movie S1). Neutrophils were rarely in B cell follicles but mainly in interfollicular zones and floor of subcapsular sinus (SCS), in close proximity to T cells (Fig. 2*C*), SCS macrophages (Fig. 2*D*), and dendritic cells (DCs) (Fig. 2*E*). To determine neutrophil dynamic behavior, we performed intravital imaging of popliteal LNs of lysozyme M-green fluorescent protein (LysM-GFP) mice (24). Neutrophils exhibited random crawling at a mean speed of 5.8 μm/min (Fig. 2 *F* and *G* and Movie S2), similar to that of LN lymphocytes (25, 26). Neutrophil numbers did not increase during imaging, suggesting their presence was not due to damage from the imaging protocol.

### Circulating Neutrophils Traffic via HEVs and Lymphatics into LNs.

**Fig. 3.**
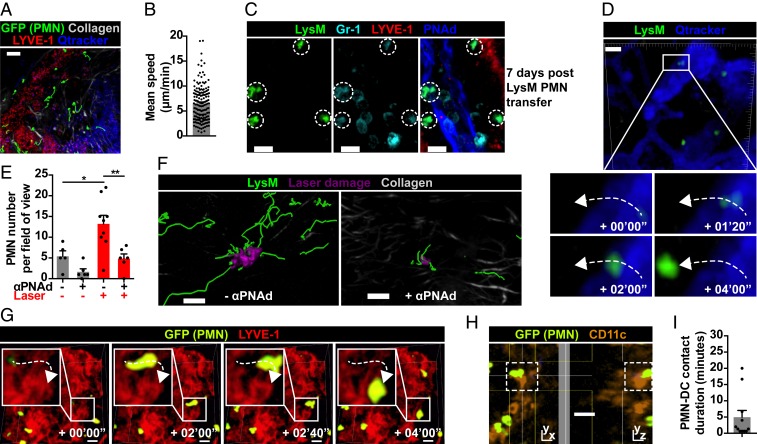
Circulating neutrophils traffic via HEVs and lymphatics into LNs. (*A*) Intravital imaging of C57BL/6 popliteal LN 24 h post i.v. GFP^+^ neutrophil transfer; movements are in green tracks. (Scale bar, 50 μm; Z stack, 80 μm.) (*B*) Mean speed of transferred neutrophils in LN; data are from 3 movies, each dot representing one cell track. (*C*) Confocal image of C57BL/6 mesenteric LN 7 d post i.v. LysM-GFP neutrophil transfer. (Scale bars, 10 μm; Z stack, 18 μm.) (*D*) Example of neutrophil trafficking from an HEV into a popliteal LN. (Scale bar, 20 μm; Z stack, 90 μm.) (*E*) Quantification of LN neutrophils pre and post laser damage; data are from 5 to 9 movies in 3 experiments per condition, each dot representing one movie; *P < 0.05 control vs. laser, **P < 0.01 laser vs. laser + anti-PNAd. (*F*) Intravital imaging of popliteal LN neutrophils (movements are in green tracks) post laser damage, without and with PNAd blockade. (Scale bars, 50 μm; Z stack, 70 μm.) (*G*) Example of neutrophil trafficking from lymphatic vessel into popliteal LN. (Scale bars, 20 μm; Z stack, 60 μm.) (*H*) Example of neutrophil–DC interaction (dashed white boxes) in popliteal LN. (Scale bar, 20 μm; Z stack, 80 μm.) (*I*) Quantification of neutrophil–DC contact duration, each dot representing one interaction. Mean ± SEM is shown.

To determine whether circulating neutrophils traffic tonically into LNs, we isolated BM GFP^+^ neutrophils for i.v. transfer into C57BL/6 mice, and examined recipient LNs by intravital imaging. At 24 h post transfer, neutrophils were present in LNs (Fig. 3*A* and Movie S3), moving with a mean speed of 6.5 μm/min (Fig. 3*B*), similar to that of native neutrophils. Surprisingly, at 4 to 5 d post transfer, we still observed motile GFP^+^ cells in LNs (Movie S4). We confirmed these were neutrophils, not macrophages with efferocytosed neutrophil fragments, by confocal Gr-1 staining 7 d post transfer (Fig. 3*C*). Even with the caveat that transferred BM-isolated neutrophils might include immature cells, these data challenge the dogma that survival of neutrophils exiting BM is limited to several hours. The rapid appearance of i.v. transferred neutrophils in LNs suggests migration from blood. We hypothesized this would require L-selectin/PNAd, involved in lymphocyte entry into LNs (14) and in neutrophil recruitment to LNs in inflammation (20, 21). Neutrophils entered LNs via HEVs in homeostasis (Fig. 3*D* and Movie S5), with a trend toward reduced basal neutrophil trafficking with PNAd blockade (mean neutrophils per field of view, 5.4 control vs. 1.7 anti-PNAd). Following laser-induced tissue damage, PNAd blockade resulted in significant but incomplete reduction in neutrophil recruitment (mean neutrophils per field of view, 13.2 control vs. 4.8 anti-PNAd, *P* < 0.01) (Fig. 3 *E* and *F* and Movie S6). As PNAd blockade did not completely abrogate neutrophil entry, we investigated whether neutrophils also enter LNs via lymphatics, and identified some neutrophils entering LNs via anti-LYVE-1–labeled lymphatics (Fig. 3*G* and Movie S7). Furthermore, intravital imaging of CD11c-YFP mice post i.v. GFP^+^ neutrophil transfer demonstrated neutrophil–DC interactions in LN cortex at baseline (*SI Appendix*, Fig. S2*A* and Movie S8); interactions were mostly brief, with a mean duration of 5 min and longest of 20 min (Fig. 3 *H* and *I* and *SI Appendix*, Fig. S2 *B* and *C*). Together, these data demonstrate that circulating neutrophils traffic into LNs via both blood and lymphatic vessels at baseline and in sterile inflammation, and may influence antigen-presenting cells such as DCs.

### Neutrophils Are Present in Human LNs and Express MHCII.

**Fig. 4.**
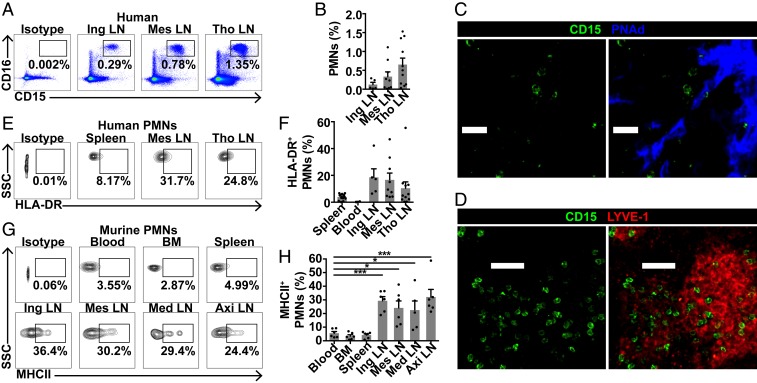
Neutrophils are present in human lymph nodes and express MHCII. (*A* and *B*) Quantification of neutrophils in human inguinal, mesenteric, and thoracic (tho) LNs with (*A*) representative plots and (*B*) % live neutrophils. (*C*) Confocal image of human thoracic LN neutrophils. (Scale bars, 20 μm; Z stack, 18 μm.) (*D*) Confocal image of human mesenteric LN neutrophils. (Scale bars, 40 μm; Z stack, 12 μm.) (*E* and *F*) MHCII expression on human LN neutrophils with (*E*) representative plots and (*F*) % MHCII^+^ neutrophils. Data are from 14 donors. (*G* and *H*) MHCII expression on unstimulated murine neutrophils with (*G*) representative plots and (*H*) % MHCII^+^ neutrophils; data are from 6 mice in 5 experiments; *P < 0.05, ***P < 0.001 vs. blood. Mean ± SEM is shown.

To determine whether neutrophils were also present in human LNs, inguinal, mesenteric, and thoracic LNs were obtained from organ donors (*SI Appendix*, Table S1) and cell suspensions were analyzed by flow cytometry, demonstrating CD15^+^CD16^+^ neutrophils in all LNs examined (Fig. 4 *A* and *B*). Confocal imaging confirmed neutrophils within LN interstitium, near HEVs and lymphatics (Fig. 4 *C* and *D*), as in murine LN neutrophils. Chemokine receptors such as CCR7 and CXCR4, and integrins including CD11b, are involved in neutrophil LN migration in inflammation (18, 21, 27). We therefore examined their baseline expression on human and murine LN neutrophils. There was little CCR7 expression, but a subset of LN neutrophils expressed CXCR4, with a higher proportion compared with blood neutrophils (*SI Appendix*, Fig. S3 *A*–*D*). CD11b expression did not differ between neutrophils from LNs and other organs (*SI Appendix*, Fig. S3 *E*–*H*). Given that neutrophils in interfollicular zones are ideally positioned to activate CD4 T cells, we next examined baseline MHCII expression in LN neutrophils; 10 to 20% of human LN neutrophils expressed MHCII, although this was variable, up to 50% in some donors (Fig. 4 *E* and *F*). Murine LN neutrophils showed significantly higher baseline MHCII expression compared with blood neutrophils (Fig. 4 *G* and *H*). These data suggest that some LN neutrophils express MHCII in homeostasis, with potential for antigen presentation.

### FcγR Cross-Linking by IC Increases Neutrophil MHCII Expression and CD4 T Cell Activation Ex Vivo.

**Fig. 5.**
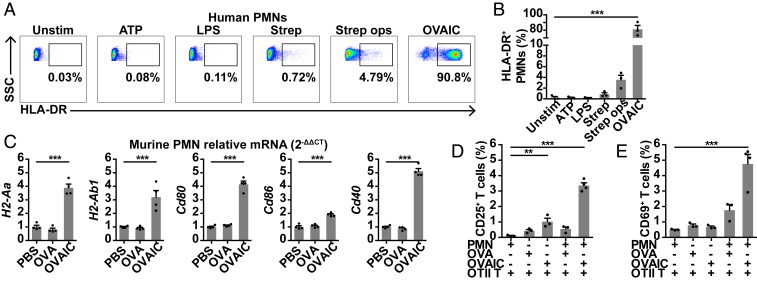
FcγR cross-linking by IC increases neutrophil MHCII expression and CD4 T cell activation ex vivo. (*A* and *B*) MHCII expression on human blood neutrophils following 1-h ex vivo stimulation with ATP, LPS, *S. pneumoniae* or OVAIC (generated with whole OVA), with (*A*) representative plots and (*B*) % MHCII^+^ neutrophils; data are from 3 donors in triplicate; ***P < 0.001 vs. unstimulated. (*C*) *H2-Aa*, *H2-Ab1*, *Cd80*, *Cd86*, and *Cd40* mRNA expression, relative to *Gapdh* (2^−ΔΔCT^), in murine BM neutrophils following 2-h ex vivo stimulation with OVA or OVAIC; 1 of 2 experiments is shown, each dot representing a triplicate; ***P < 0.001 OVAIC vs. PBS. (*D* and *E*) Expression of (*D*) CD25 and (*E*) CD69 on OTII CD4 T cells following coculture with OVA- or OVAIC-stimulated neutrophils; 1 of 2 experiments is shown; **P < 0.01, ***P < 0.001 vs. PMN + OTII T control. Mean ± SEM is shown.

To further characterize signals that influence neutrophil MHCII expression, we stimulated human blood neutrophils ex vivo with ATP (DAMP), lipopolysaccharide (LPS; TLR4 agonist), *Streptococcus pneumoniae* (TLR2 agonist), and whole ovalbumin (OVA)–IgG IC (OVAIC; FcγR cross-linking), as well as CCL19 (CCR7 ligand), CXCL4, leukotriene B4 (LTB4), and fMLP. OVAIC significantly up-regulated neutrophil MHCII expression, in contrast to other stimuli (Fig. 5 *A* and *B* and *SI Appendix*, Fig. S4), and up-regulated neutrophil CCR7 and CD11b (18, 21, 27) expression (*SI Appendix*, Fig. S5). Ex vivo OVAIC stimulation of murine BM neutrophils also up-regulated MHCII (*H2-Aa* and *H2-Ab1*) and costimulatory molecules (*Cd80*, *Cd86*, and *Cd40*) (Fig. 5*C*). Coculture of murine OVAIC-stimulated neutrophils and OVA-specific OTII CD4 T cells resulted in increased expression of CD25 and CD69, early markers of T cell activation (Fig. 5 *D* and *E* and *SI Appendix*, Fig. S6). These data demonstrate that IC induces up-regulation of MHCII and costimulatory molecules, leading to increased CD4 T cell activation ex vivo.

### Neutrophils Phagocytose Systemic and LN IC In Vivo.

**Fig. 6.**
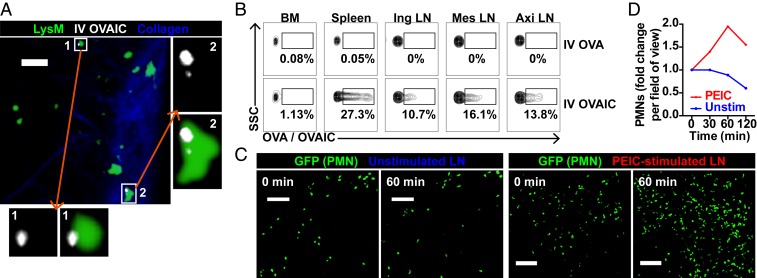
Neutrophils phagocytose systemic and LN IC in vivo. (*A*) Intravital imaging of LysM-GFP popliteal LN 2 h post i.v. OVAIC administration, with *Insets* showing OVAIC^+^ neutrophils. (Scale bar, 30 μm; Z stack, 40 μm.) (*B*) Flow cytometry plots of BM, spleen, and LN neutrophils 1 h following i.v. OVA or OVAIC administration to a LysM-GFP mouse. (*C*) Intravital imaging of popliteal LN neutrophils 0- and 60-min unstimulated and following local PEIC stimulation. (Scale bars,100 μm; Z stack, 80 μm.) (*D*) Neutrophil recruitment quantified as fold change from baseline of cells per field of view.

We next challenged neutrophils in vivo with IC, systemically or locally. One to two hours post i.v. OVAIC administration, we identified IC-positive neutrophils in peripheral LNs by intravital imaging (Fig. 6*A*, *SI Appendix*, Fig. S7*A*, and Movie S9) and flow cytometry (Fig. 6*B*). This suggests that neutrophils carry systemic IC to peripheral LNs, with the potential to initiate adaptive immune responses. We also generated IC in vivo by i.p. anti-phycoerythrin (PE) and s.c. PE administrations, with IC formed within draining LNs (28); this resulted in rapid recruitment of neutrophils, with some being IC-positive (Fig. 6 *C* and *D*, *SI Appendix*, Fig. S7*B*, and Movie S10). These data suggest that neutrophils can take up IC both in circulation and in LNs, potentially promoting MHCII expression and antigen presentation.

## Discussion

Our study demonstrates that neutrophils are present in unstimulated LNs under homeostatic conditions, in areas that enable them to interact with T cells, DCs, and SCS macrophages. Circulating neutrophils traffic into LNs via HEVs and lymphatic vessels, the former process involving PNAd, and neutrophils interact with DCs in LNs at baseline. Both human and murine LN neutrophils express MHCII, and ex vivo stimulation of human blood and murine BM neutrophils with IgG IC results in up-regulation of MHCII and costimulatory molecules, leading to increased CD4 T cell activation ex vivo. In vivo, neutrophils are capable of early uptake of systemic IC into peripheral LNs.

Neutrophil recruitment to draining LNs has been shown to occur following local infectious or inflammatory challenges via HEVs (19–21) and lymphatics (18, 21). Some of these studies demonstrated LN neutrophils at baseline (18, 20), as did a study of aged mice (29). However, these investigators did not use methods to definitively distinguish circulating from tissue neutrophils, as we have done. Casanova-Acebes et al. (22) recently showed, using parabiosis, that circulating neutrophils migrate into most tissues, including spleen, lung, intestine, and skin, with varying diurnal patterns at steady state. Our study adds to this description, characterizing in detail the number, anatomical location, and in vivo dynamic behavior of LN neutrophils in different body regions in homeostasis. We confirmed that neutrophils trafficked into LNs in the absence of perturbation and that this was not limited to mice, with a similar distribution of neutrophils in human LNs. To ensure our study accurately assessed unstimulated LNs, we used young female mice with minimal handling prior to LN harvest. Human LNs were obtained from organ donors who might have a degree of systemic inflammation, with a few infections, but the majority had no specific insult in the territories drained by the LNs. For transfer experiments, we isolated neutrophils by negative magnetic selection and transferred cells immediately post isolation to minimize time spent ex vivo.

Our observation of the presence of LN neutrophils up to 7 d post i.v. transfer challenges the dogma that neutrophils are short-lived cells. For transfer experiments, we isolated neutrophils from BM, which provides much higher yields compared with blood but contains some immature cells. Isolated BM LysM-GFP neutrophils transferred i.v. into wild-type mice have a circulatory half-life of 8 h (30). Although a previous study has shown isolated BM neutrophils to be functionally mature (31), up to 10% of isolated BM neutrophils may be immature, and these may contribute to the longer-lived neutrophils observed in LNs post transfer. In vivo labeling studies have shown variable estimates of circulating neutrophil half-life partly due to different study methodologies, such as labeling of whole blood or isolated neutrophils, and different methods and durations of labeling (3–7, 32). Agents such as LPS delay neutrophil apoptosis in vitro (33), and inflammatory alveolar neutrophils show prolonged survival when cultured ex vivo (34). It may be that prosurvival signals or cellular interactions encountered by LN neutrophils increase their longevity compared with blood neutrophils, but this requires further investigation.

We found that circulating neutrophils were capable of delivering IC from blood to LNs, observing IC-containing neutrophils in LNs within 1 h of systemic IC administration. This suggests that neutrophils may act as circulatory sentinels in homeostasis, performing immune surveillance in the bloodstream, gathering systemic antigens, and rapidly delivering them to LNs. We did not directly determine in vivo whether these IC-carrying neutrophils internalize and present IgG-opsonized antigens to CD4 T cells in LNs, but we showed that LN neutrophils localized to the interfollicular zone, a critical area for T cell activation, and a subset of LN neutrophils showed high MHCII expression. This, with the finding that IC-stimulated neutrophils up-regulated MHCII and costimulatory molecules and activated CD4 T cells ex vivo, is consistent with the conclusion that circulating IgG-opsonized antigens sampled by neutrophils may be directly presented to CD4 T cells in LNs. Other studies have also observed that neutrophils activate antigen-specific CD4 T cells in vitro (11, 12). Together, these data provide evidence of neutrophil function beyond innate immunity.

To determine signals governing neutrophil recruitment to LNs, we used PNAd antibody blockade to inhibit neutrophil migration via HEVs but did not concomitantly block lymphatic migration. Previous studies have shown CCR7 and CXCR4 to be involved in neutrophil lymphatic migration during inflammation (21, 27). Consistent with this, we found higher surface CXCR4 expression on LN neutrophils at baseline. We examined surface CCR7 expression, but found no difference between circulating and LN neutrophils. This may be due to CCR7 internalization following ligand engagement upon entry into LNs. Notably, ex vivo IC-stimulated neutrophils up-regulated MHCII, CCR7, and CD11b, but the signals driving basal LN neutrophil MHCII expression, without IC stimulation, are unclear. We tested the chemoattractants CCL19, CXCL4, LTB4, and fMLP, but none affected neutrophil MHCII expression. Since the integrin Mac-1 (CD11b/CD18) facilitates neutrophil migration and may mediate outside-in signaling via both PI3K-Akt and p38 MAPK-ERK (35), pathways also activated upon FcγR cross-linking by IC, this represents a potential mechanism for basal neutrophil MHCII expression.

In summary, our study challenges the perception that neutrophil patrol is limited to the circulation in homeostasis, adding LNs to their routine surveillance territory. Our data also suggest a broader functional remit for neutrophils as cells that sample antigens within the circulation and traffic immunological information to LNs, potentially impacting adaptive immunity.

## Materials and Methods

### Mice.

C57BL/6 and LysM-GFP mice (*SI Appendix*, Fig. S8) were maintained in specific pathogen-free conditions, and experiments were approved in accordance with Animals (Scientific Procedures) Act 1986 UK.

### Murine LNs.

Female mice aged 8 to 10 wk were used and i.v. anti-CD45 was administered within 1 min of euthanization.

### Murine Neutrophil Transfer.

BM GFP^+^ neutrophils were isolated by magnetic negative selection, with 98% purity (*SI Appendix*, Fig. S9), and 2 × 10^6^ neutrophils per mouse were transferred intravenously.

### Human LNs and Neutrophil Isolation.

Whole LNs were removed from deceased organ donors. Blood neutrophils were isolated as previously described (36). Informed consent was obtained. All human blood and tissue samples were deidentified prior to use. Ethical approval was obtained from the East of England Research Ethics Committee (12/EE/0446, 15/EE/0152), and experiments were performed in accordance with the Declaration of Helsinki.

### IC Stimulation.

Whole OVA was incubated with rabbit anti-OVA (1:10) at 37 °C for 1 h to generate IC. For in vivo stimulation, OVAIC was injected intravenously, and PEIC was generated locally by i.p. anti-PE and s.c. PE to the hock.

### Intravital and Confocal Microscopy.

Popliteal LN intravital imaging was performed with a Chameleon Vision-S tunable Ti:sapphire laser and Leica TCS SP8 microscope at 36 °C. LNs were fixed and frozen sections were used for confocal imaging. Images were processed using Bitplane Imaris and ImageJ (NIH). Statistical analysis was with unpaired *t* tests or one-way ANOVA and Holm–Sidak correction; *P* < 0.05 was statistically significant. See *SI Appendix* for further details.

## Supplementary Material

Supplementary File

Supplementary File

Supplementary File

Supplementary File

Supplementary File

Supplementary File

Supplementary File

Supplementary File

Supplementary File

Supplementary File

Supplementary File
