# Supplementary material for: Phenotypically distinct neutrophils patrol uninfected human and mouse lymph nodes
Source: Proc Natl Acad Sci U S A. 2019 Sep 4;116(38):19083–9. doi: 10.1073/pnas.1905054116 (PMC6754587; doi:10.1073/pnas.1905054116)
Supplement: Supplementary File [file pnas.1905054116.sapp.pdf]

Supporting Information

Phenotypically distinct neutrophils patrol uninfected human and mouse lymph nodes

Laurence S.C. Lok, Thomas W. Dennison, Krishnaa M. Mahbubani, Kourosh Saeb-Parsy, Edwin R. Chilvers, Menna R. Clatworthy

Materials and Methods

Mice

C57BL/6 mice were purchased from Charles River Laboratories UK. LysM-GFP mice were a gift from Sussan Nourshargh (Queen Mary University London); neutrophils are GFP-bright, distinguishing them from GFP-intermediate monocytes and macrophages (SI Appendix Fig. S8). Mice were maintained in specific pathogen-free conditions, and all experimental procedures were approved in accordance with the Animals (Scientific Procedures) Act 1986 UK.

Murine unstimulated LN analysis

Female mice aged 8 to 10 weeks were used. An intravenous CD45 antibody (1 µg) was administered immediately prior to euthanization (less than 1 minute) to ensure that leukocyte labelling was limited to cells within the vasculature. LNs were harvested and mechanically disaggregated for flow cytometry, or fixed and frozen sections generated for confocal microscopy.

Murine neutrophil isolation and cell transfer

Neutrophils were isolated from the BM of LysM-GFP mice using MACS magnetic negative selection as per manufacturer's instructions (Miltenyi Biotec); 98% of isolated GFP$^+$ cells were Ly6G$^+$ (SI Appendix Fig. S9). 2 x 10^6 neutrophils per mouse were injected intravenously into C57BL/6 recipients for intravital imaging. For PNAd blockade, mice were injected intraperitoneally with anti-PNAd (200 µg) 4-6 hours prior to neutrophil transfer, and a second dose 1 hour prior to intravital imaging.

Human LN analysis and neutrophil isolation

Whole human LNs were removed from deceased transplant organ donors. Human blood neutrophils were isolated as previously described (1) for *ex vivo* stimulation. All human blood and tissue samples were de-identified prior to use in the study. Ethical approval was obtained from East of England Research Ethics Committee (references 12/EE/0446, 15/EE/0152), and experiments were performed in accordance with the Declaration of Helsinki.

Antibodies and flow cytometry

Antibodies were purchased from BioLegend, eBioscience or Thermo Fisher. Anti-mouse antibodies included: Live/dead Aqua, Ly6G (1A8)-PB or A647, Gr-1 (RB6-8C5)-PB, CD11b-PB or BV605, CD45-eF450, B220-PB or A647, CD3-BV605 or PE, MHCII-A700, LYVE-1-PE, PNAd (MECA-79)-biotin, streptavidin-A647, CD169-A647, CD25-PerCP.Cy5.5, CD69-BV785, CD4-A780. Rabbit polyclonal anti-GFP-A488 was used for confocal staining. Anti-human antibodies included: CD16-PB, BV650 or APC.eF780, HLA-DR-PB or PerCP.eF710, CCR7-FITC, CD15-FITC, BV650, or A700, PNAd-PE, LYVE-1-APC. For flow cytometry antibodies were used at 1:200; data were acquired using LSRFortessa flow cytometer and FACSDiva software, and analyzed using FlowJo software.

IC stimulation

IC was generated *in vitro* by incubating A647-conjugated whole OVA with rabbit anti-OVA (1:10 ratio) at 37°C for 1 hour. OVAIC (5 µg/ml OVA) was used to stimulate human blood neutrophils for 1 hour followed by flow cytometry, or murine BM neutrophils for 2 hours followed by reverse-transcriptase polymerase chain reaction (RT-PCR) using primers for *H2-Aa*, *H2-Ab1*, *Cd80*, *Cd86* and *Cd40*. For co-culture experiments, OVA or OVAIC-stimulated neutrophils were incubated with OTII CD4 T cells (2:1) for 48 hours prior to staining for flow cytometry. For *in vivo* stimulation experiments, OVAIC was injected intravenously (5 µg OVA / mouse); PEIC was generated locally *in vivo* by intraperitoneal administration of 2 mg rabbit anti-PE followed by subcutaneous footpad injection of 15 µg PE.

Confocal microscopy

LNs were fixed in 1% paraformaldehyde, dehydrated in 30% sucrose, and frozen in optimal cutting temperature (OCT) compound. 30-50 µm sections were stained overnight at 4°C in a

buffer containing 0.1 M TRIS, 1% bovine serum albumin, 1% mouse serum and 0.3% Triton X-100. Secondary antibodies were applied for 30 minutes before mounting with Vectashield medium. Images were acquired using a Leica TCS SP8 microscope and 40x oil immersion objective, with optical sections under 2 μm.

Intravital microscopy

Intravenous Qtracker655 (10-20 μl) and subcutaneous anti-LYVE-1-PE (5 μl to hock) were used to label blood and lymphatic vessels respectively. Popliteal LN was exposed and intravital imaging performed with a Chameleon Vision-S tuneable Ti:Sapphire multi-photon laser and Leica TCS SP8 microscope with the animal kept at 36°C throughout. Sequential excitation was used, with two-photon excitation at 920 nm for GFP, collagen second harmonics, PE and Qtracker, and single photon excitation at 633 nm for A647. For some experiments focal laser damage was achieved by two-photon excitation of a confined area at 860 nm and 90% power for 1 minute. Movies were acquired using a 25x water objective, with one Z stack every 40 seconds and 2 μm optical sections.

Image processing and data analysis

Intravital movies and confocal images were processed using Bitplane Imaris and ImageJ (NIH). Cells were tracked using the ‘surfaces’ function and dynamic parameters such as mean speeds generated. Time-lapse movies were recorded at 10 frames per second (fps), or at slower rates (as indicated) for clarity of presentation. Data were analyzed using GraphPad Prism, with statistical analysis performed using unpaired t-tests or one-way ANOVA and Holm-Sidak correction for between-group multiple comparisons. Data are shown as mean ± SEM unless otherwise indicated. $p < 0.05$ was considered statistically significant.

Supplementary Figures

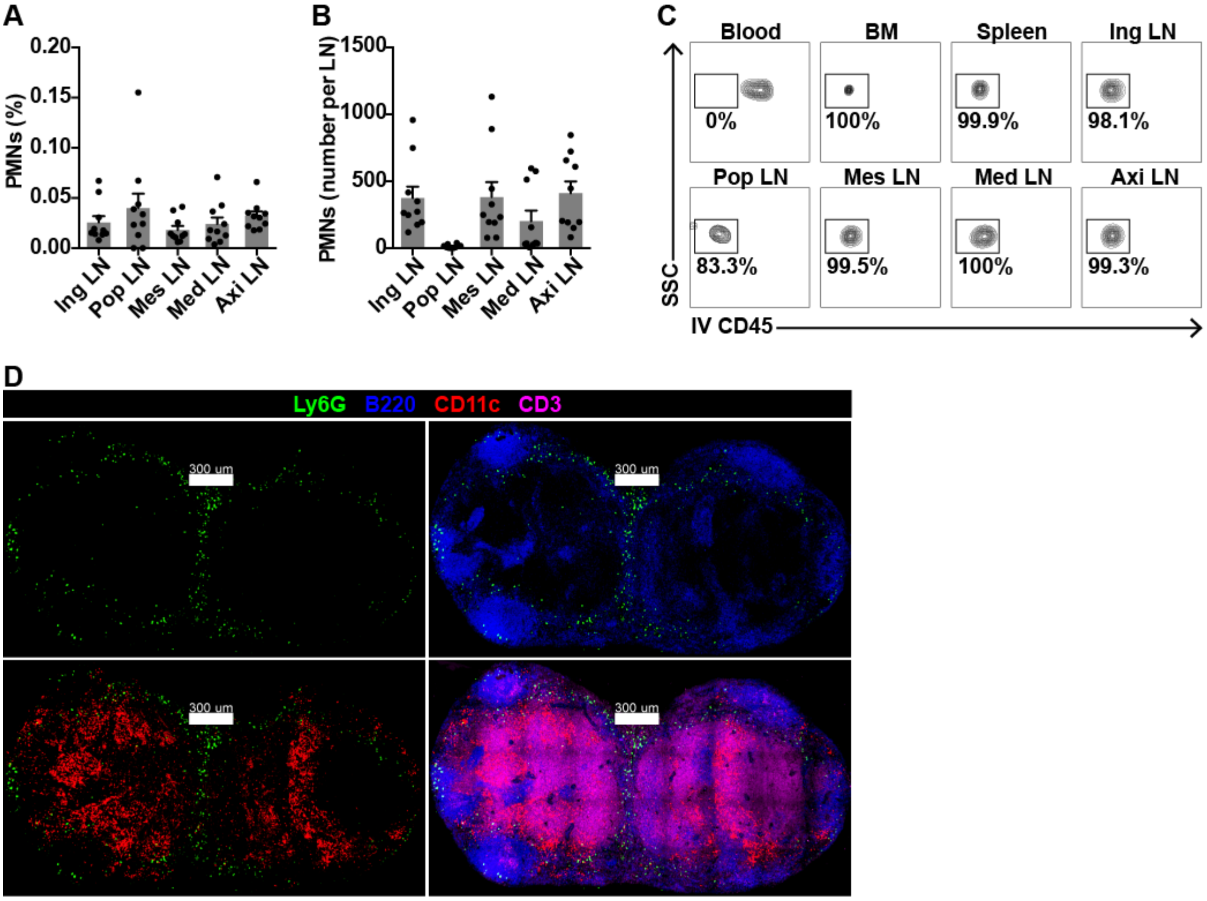

Figure S1. Quantification of neutrophil numbers in unstimulated LNs. Flow cytometry quantification of inguinal (ing), popliteal (pop), mesenteric (mes), mediastinal (med) and axillary (axi) LNs showing (A) neutrophil numbers as % live cells, and (B) absolute neutrophil numbers per LN; data from 10 mice in 7 experiments. Mean ± SEM shown. (C) Representative flow cytometry plots showing live intravenous $CD45^{-}$ (extravascular) neutrophils. (D) Confocal image of unstimulated C56BL/6 inguinal LN; scale bar 300 μm, Z stack 5 μm.

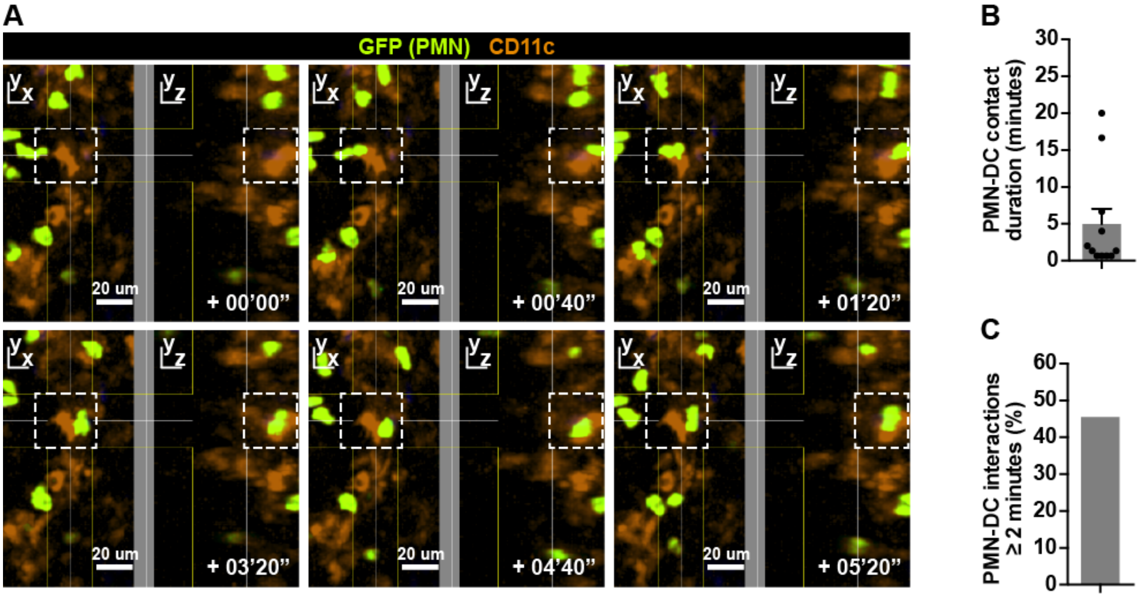

Figure S2. Neutrophil-DC interactions in LN at baseline *in vivo*. (A) Intravital imaging of popliteal LN showing example of interaction (dotted white boxes) between GFP$^+$ neutrophil and CD11c$^+$ DC; image shown in two planes, scale bar 20 µm, Z stack 80 µm. (B-C) Quantification of (B) duration of neutrophil-DC contact time in minutes and (C) % of neutrophil-DC interactions lasting ≥ 2 minutes; each dot represents one interaction. Mean ± SEM shown.

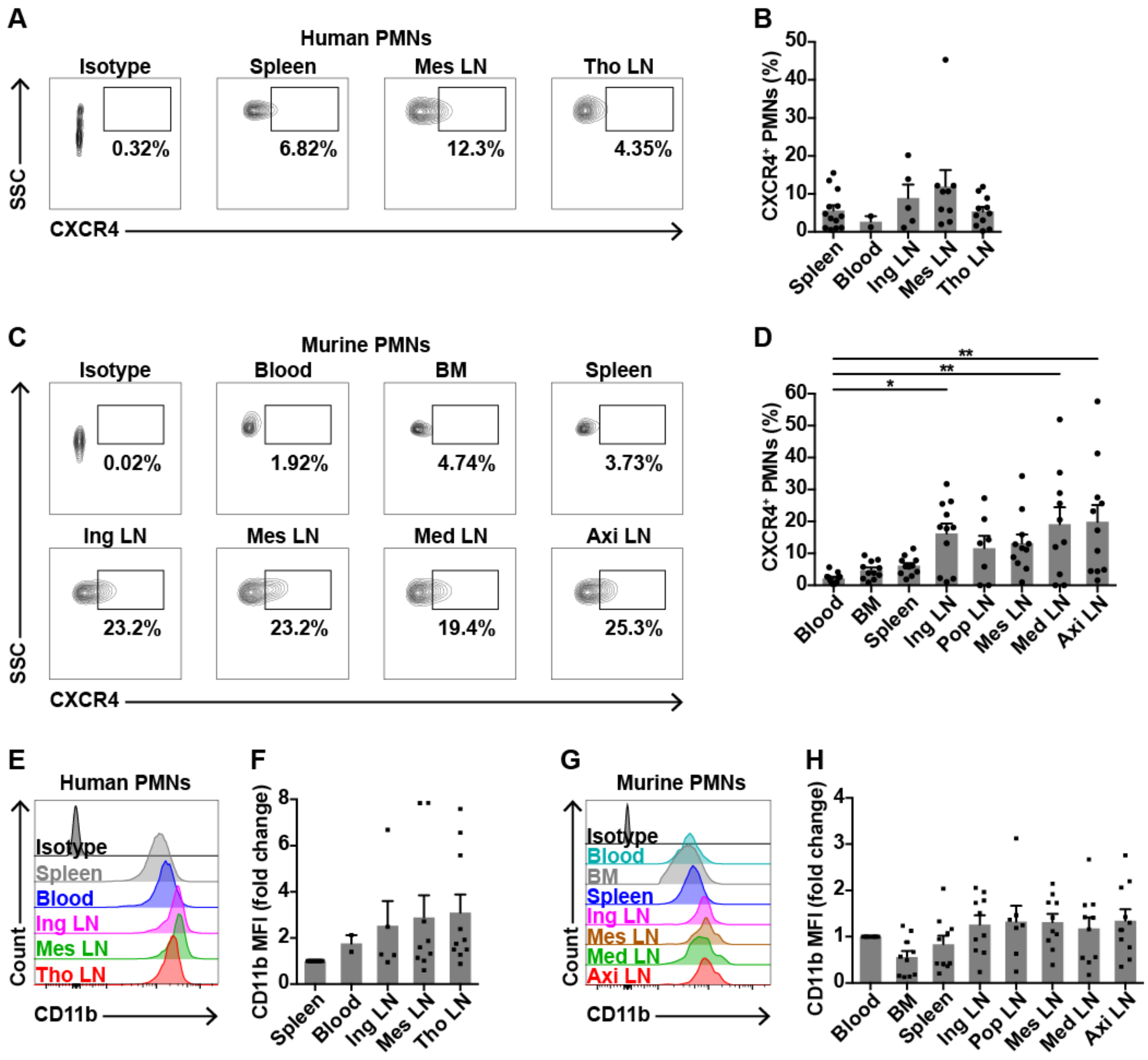

Figure S3. CXCR4 and CD11b surface expression on human and murine LN neutrophils. (A-D) CXCR4 expression on (A-B) human and (C-D) unstimulated murine LN neutrophils, with representative plots and % $CXCR4^+$ neutrophils shown; **p* < 0.05, ***p* < 0.01 *vs* blood. (E-H) CD11b expression on (E-F) human and (G-H) unstimulated murine LN neutrophils, with representative plots and neutrophil CD11b MFI shown. Human data from 14 donors; murine data from 6 unstimulated mice in 5 experiments. Mean ± SEM shown.

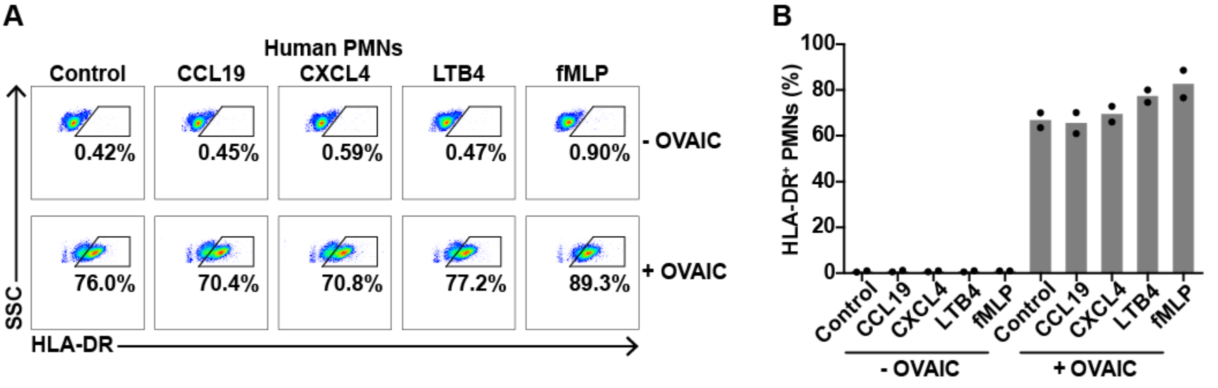

Figure S4. MHCII surface expression on neutrophils in response to chemoattractants *ex vivo*. Isolated neutrophils were stimulated *ex vivo* for 1 hour with the chemoattractants CCL19, CXCL4, LTB4 and fMLP, alone or in combination with OVAIC, followed by staining for MHCII expression. (A) Representative plots and (B) % MHCII^{+} neutrophils shown; data from 2 donors in triplicates. Means shown.

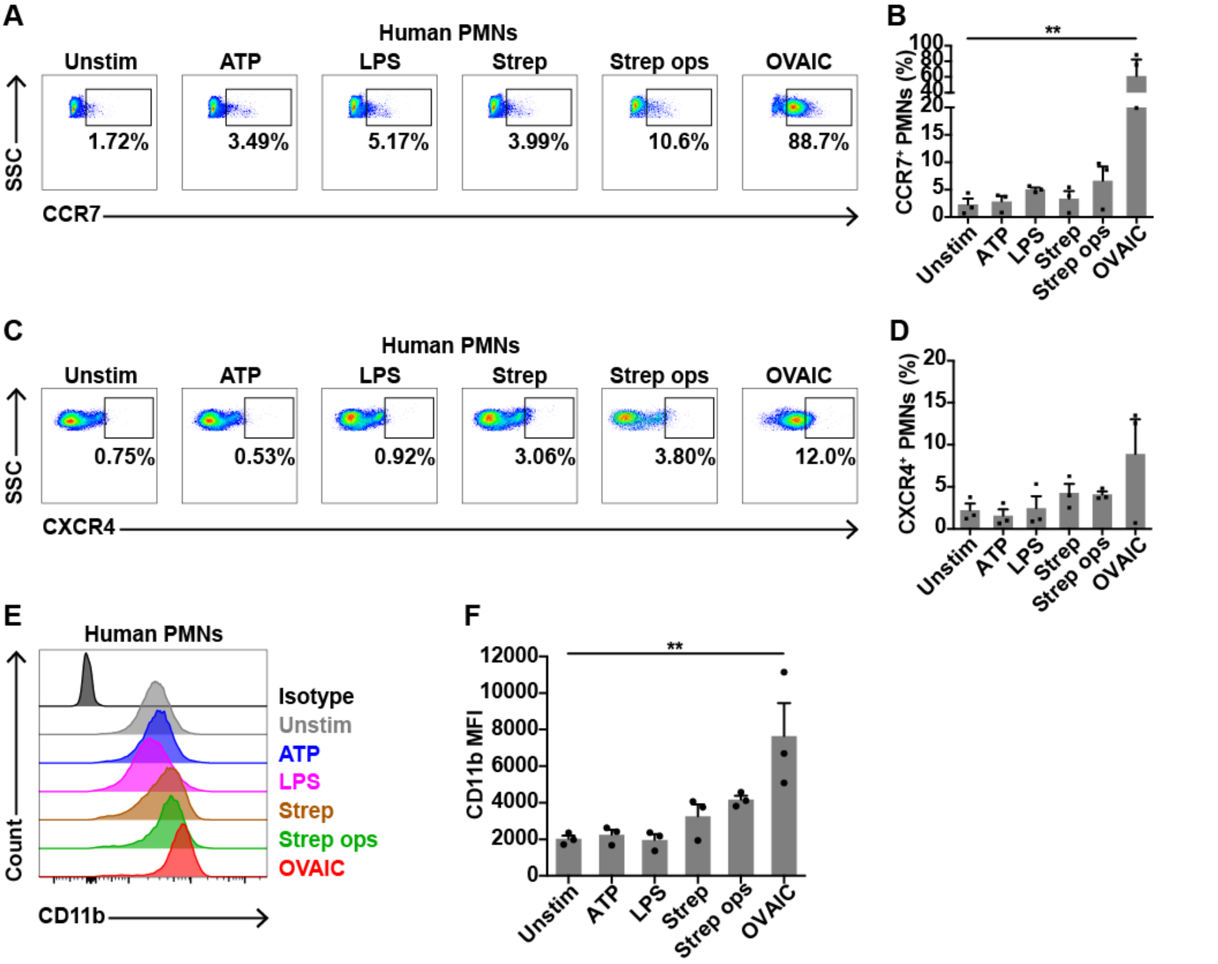

Figure S5. CCR7, CXCR4 and CD11b surface expression on neutrophils following *ex vivo* stimulation. Isolated human neutrophils were stimulated *ex vivo* for 1 hour with ATP, LPS, unopsonised *Streptococcus pneumoniae* (Strep), opsonised *Streptococcus pneumoniae* (Strep ops) or OVAIC, followed by staining for flow cytometry. (A-B) Neutrophil CCR7 expression, with (A) representative plots and (B) % $CCR7^{+}$ neutrophils. (C-D) Neutrophil CXCR4 expression, with (C) representative plots and (D) % $CXCR4^{+}$ neutrophils. (E-F) Neutrophil CD11b expression, with (E) representative plots and (F) CD11b MFI. Data from 3 donors in triplicates; $^{**}p < 0.01$ OVAIC *vs* unstimulated. Mean ± SEM shown.

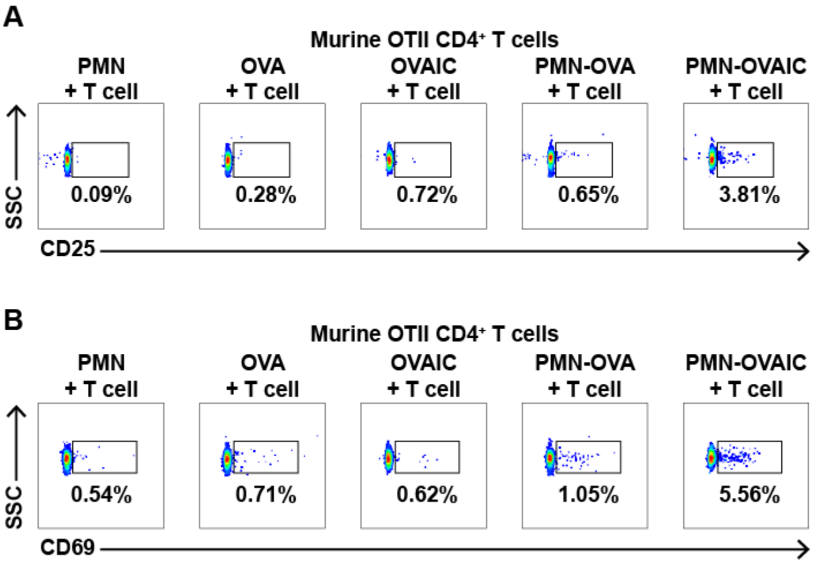

Figure S6. OVAIC-stimulated neutrophils increase CD4 T cell activation *ex vivo*. Representative plots showing expression of (A) CD25 and (B) CD69 on OTII CD4 T cells following co-culture with OVA- or OVAIC-stimulated neutrophils or controls.

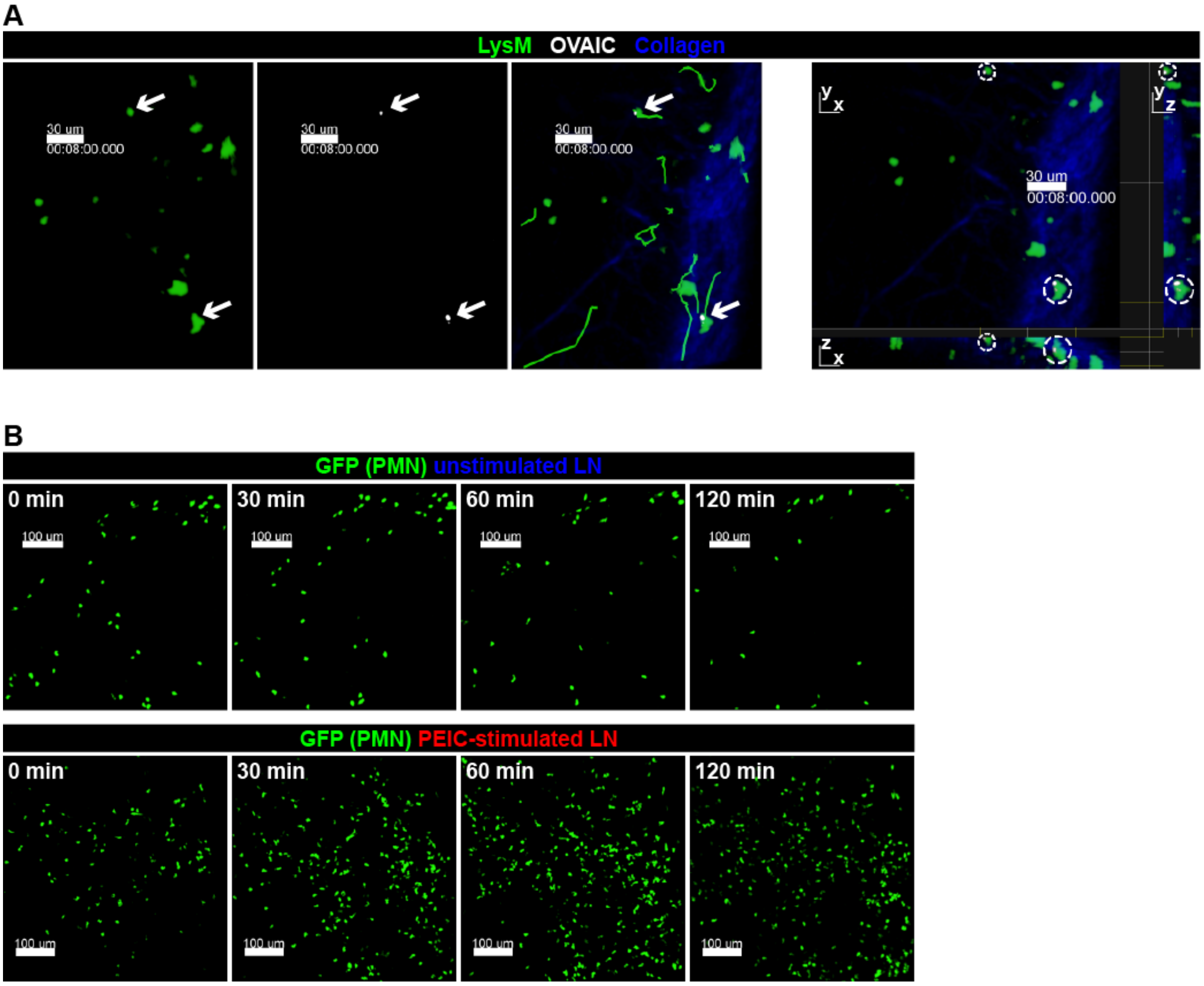

Figure S7. Neutrophils phagocytose systemic and LN IC *in vivo*. (A) Intravital imaging of LysM-GFP popliteal LN 2 hours post intravenous OVAIC administration, with neutrophil tracks in green, right panel showing image in 3 planes, and white arrows and dotted circles showing OVAIC^{+} neutrophils; scale bar 30 µm, Z stack 40 µm. (B) Intravital imaging of neutrophil recruitment to popliteal LNs 0, 30, 60 and 120 minutes unstimulated and following local PEIC stimulation; scale bar 100 µm, Z stack 80 µm.

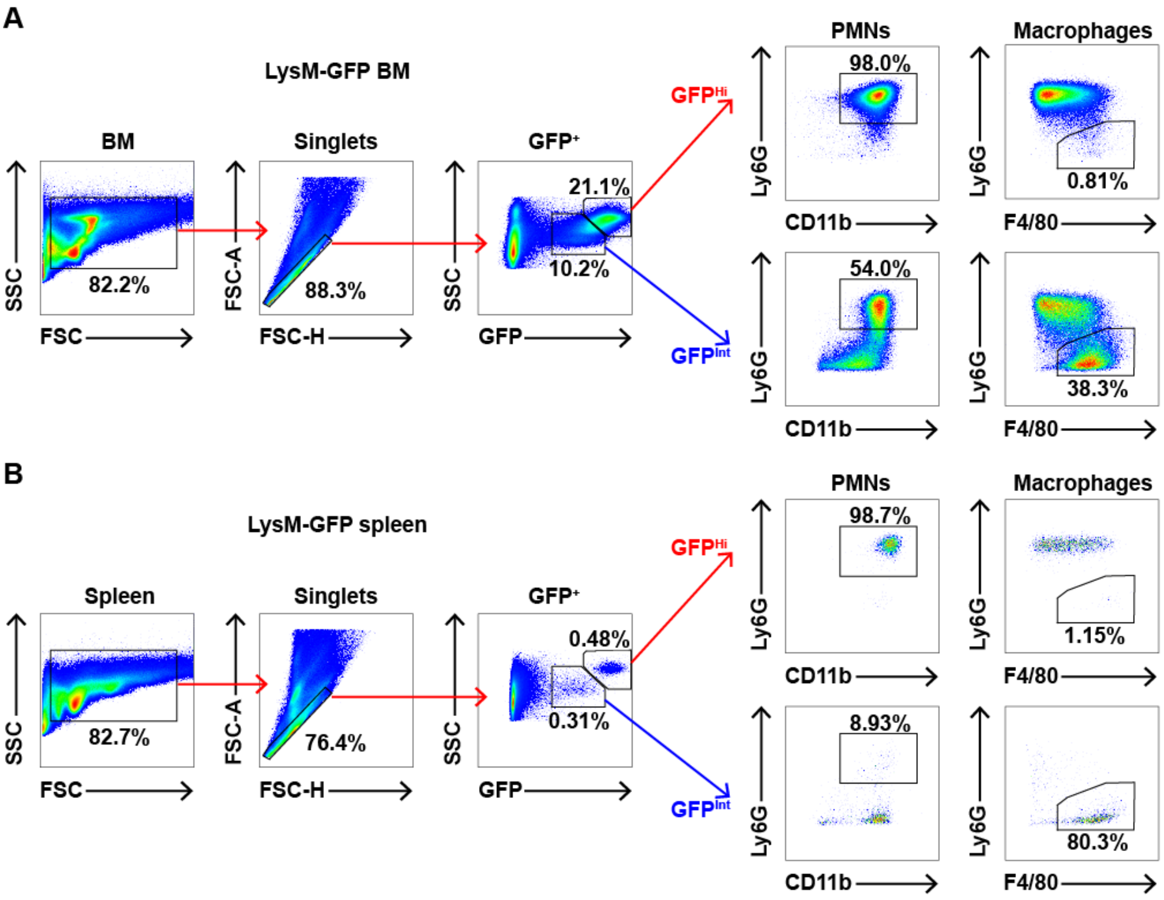

Figure S8. Specificity of LysM-GFP mouse for neutrophils in tissues. Flow cytometry plots showing proportions of neutrophils (Ly6G$^+$CD11b$^+$) and macrophages (Ly6G$^-$F4/80$^+$) in GFP-high (GFP^{Hi}) and GFP-intermediate (GFP^{Int}) cell populations in (A) BM and (B) spleen of unstimulated LysM-GFP mouse.

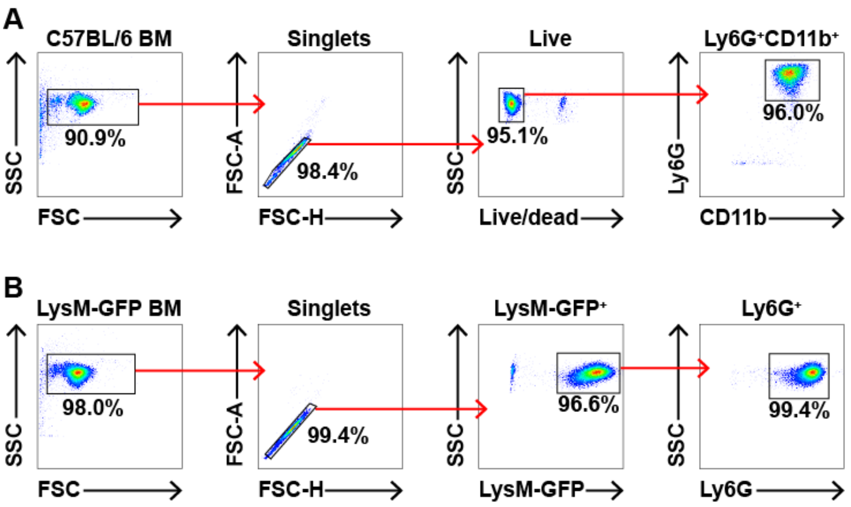

Figure S9. Purity of murine neutrophil isolation. Flow cytometry plots showing neutrophil purity post isolation from BM of (A) C57BL/6 mouse and (B) LysM-GFP mouse.

Supplementary Table

Age	Sex	Donor type	Cause of death	Known infections	Co-morbidities
69	F	DBD	Intracranial haemorrhage	None	Hypercholesterolaemia
71	M	DBD	Intracranial haemorrhage	None	Hypertension, ischaemic heart disease, asthma
63	M	DCD	Intracranial haemorrhage	None	High alcohol intake
30	M	DBD	Cerebral oedema	Cerebral abscess	Diabetes
76	M	DBD	Intracranial haemorrhage	None	None
77	M	DCD	Subdural haematoma post RTC	Fever of unknown source	Diabetes, peripheral vascular disease, hypertension, depression
27	F	DBD	RTC	None	None
23	M	DCD	Hypoxic brain injury post RTC	None	None
10	F	DBD	Glioma with intracranial hypertension	None	Crohn’s disease
22	M	DCD	Cardiac arrest post heroin overdose	Possible aspiration pneumonia	None
64	M	DCD	Acute respiratory distress syndrome	Bacteraemia	Diabetes
59	M	DCD	Hypoxic brain injury	None	None
78	F	DBD	Intracranial haemorrhage	None	Hypertension
52	F	DBD	Intracranial haemorrhage	Lobar pneumonia	Psoriasis

Table S1. Summary of human organ donor characteristics. Data from 13 spleen, 2 blood, 5 inguinal LN, 9 mesenteric LN and 11 thoracic LN samples from a total of 14 donors. Age in years. M, male; F, female; DBD, donation after brainstem death; DCD, donation after circulatory death; RTC, road traffic collision.

Movie Legends

Movie S1. Neutrophils are present in unstimulated LN. Confocal image of inguinal LN of unstimulated LysM-GFP mouse, showing location of neutrophils in LN relative to blood and lymphatic vessels; scale bar 15 to 300 μm, Z stack 10 μm.

Movie S2. LN neutrophils are motile at baseline. Intravital two-photon imaging of unstimulated LysM-GFP popliteal LN; scale bar 50 μm, Z stack 50 μm, time-lapse movie over 30 minutes at 10 fps.

Movie S3. Circulating neutrophils traffick tonically into LN. Intravital imaging of C57BL/6 popliteal LN 24 hours following intravenous transfer of GFP^+ neutrophils; scale bar 30 to 50 μm, Z stack 80 μm, time-lapse movie over 90 minutes at 10 fps.

Movie S4. Motile neutrophils are present in LN 4 days post transfer. Intravital imaging of C57BL/6 popliteal LN 4 days following intravenous transfer of LysM-GFP neutrophils; scale bar 30 μm, Z stack 80 μm, time-lapse movie over 45 minutes at 10 fps.

Movie S5. Neutrophils traffick into LN via HEVs in homeostasis. *In vivo* examples (white arrows) of neutrophils trafficking from HEV into popliteal LN; scale bar 20 μm, Z stack 90 μm, time-lapse movie over 20 minutes at 3 fps.

Movie S6. PNAd blockade impairs neutrophil recruitment to LN. Intravital imaging of recruitment of intravenously transferred LysM-GFP neutrophils (movements in green tracks) to popliteal LN following local laser damage, without PNAd blockade (- αPNAd) and with PNAd blockade (+ αPNAd); scale bar 50 μm, Z stack 70 μm, time-lapse movies over 45 minutes per condition at 10 fps.

Movie S7. Neutrophils traffick into LN via lymphatics in homeostasis. *In vivo* example (white arrow) of neutrophil trafficking from lymphatic vessel into popliteal LN; scale bar 20 μm, Z stack 60 μm, time-lapse movie over 5 minutes at 1.7 fps.

Movie S8. Neutrophils interact with DCs in LN cortex at baseline. Intravital imaging of popliteal LN of CD11c-YFP mouse 24 hours following intravenous GFP^+ neutrophil transfer, showing neutrophil-DC interactions (white boxes); scale bar 20 μm, Z stack 40 to 80 μm, two time-lapse movies over 15 to 20 minutes at 3 fps.

Movie S9. Neutrophils phagocytose systemic IC and migrate into LN. Intravital imaging of popliteal LN of LysM-GFP mouse 2 hours following intravenous OVAIC (white) administration, showing $OVAIC^+$ neutrophils (white arrows); scale bar 30 μm, Z stack 40 μm, two time-lapse movies each over 12 minutes at 2 fps.

Movie S10. Neutrophils phagocytose local IC within LN. Intravital imaging of popliteal LN following local PEIC stimulation, showing PEIC uptake (yellow) by neutrophils; scale bar 15 μm, Z stack 60 μm, time-lapse movie over 20 minutes at 2 fps.

References

1. L.S.C. Lok *et al*. Effects of tocilizumab on neutrophil function and kinetics. *Eur J Clin Invest*. **47(10)**, 736–745 (2017).
